# A Research Protocol to Study the Priming Effects of Breathing Low Oxygen on Enhancing Training-Related Gains in Walking Function for Persons With Spinal Cord Injury: The BO_2_ST Trial

**DOI:** 10.1089/neur.2023.0036

**Published:** 2023-11-06

**Authors:** William M. Muter, Linda Mansson, Christopher Tuthill, Shreya Aalla, Stella Barth, Emily Evans, Kelly McKenzie, Sara Prokup, Chen Yang, Milap Sandhu, W. Zev Rymer, Victor R. Edgerton, Parag Gad, Gordon S. Mitchell, Samuel S. Wu, Guogen Shan, Arun Jayaraman, Randy D. Trumbower

**Affiliations:** ^1^ Spaulding Rehabilitation Hospital, Charlestown, Massachusetts, USA.; ^2^ Department of Physical Medicine and Rehabilitation, Harvard Medical School, Boston, Massachusetts, USA.; ^3^ Shirley Ryan AbilityLab, Max Nader Center for Rehabilitation Technologies and Outcomes Research, Chicago, Illinois, USA.; ^4^ Department of Integrative Biology and Physiology, University of California–Los Angeles, Los Angeles, California, USA.; ^5^ SpineX Inc., Northridge, California, USA.; ^6^ Department of Biostatistics, University of Florida, Gainesville, Florida, USA.; ^7^ UMass Chan Medical School, University of Massachusetts, Worcester, Massachusetts, USA.; ^8^ Department of Physical Therapy, Boston University, Boston, Massachusetts, USA.; ^9^ Department of Physical Therapy, University of Florida, Gainesville, Florida, USA.

**Keywords:** electrical stimulation, hypoxia, neurological rehabilitation, neuromodulation, plasticity, spinal cord trauma, walking

## Abstract

Brief episodes of low oxygen breathing (therapeutic acute intermittent hypoxia; tAIH) may serve as an effective plasticity-promoting primer to enhance the effects of transcutaneous spinal stimulation-enhanced walking therapy (WALK_tSTIM_) in persons with chronic (>1 year) spinal cord injury (SCI). Pre-clinical studies in rodents with SCI show that tAIH and WALK_tSTIM_ therapies harness complementary mechanisms of plasticity to maximize walking recovery. Here, we present a multi-site clinical trial protocol designed to examine the influence of tAIH + WALK_tSTIM_ on walking recovery in persons with chronic SCI. We hypothesize that daily (eight sessions, 2 weeks) tAIH + WALK_tSTIM_ will elicit faster, more persistent improvements in walking recovery than either treatment alone. To test our hypothesis, we are conducting a placebo-controlled clinical trial on 60 SCI participants who randomly receive one of three interventions: tAIH + WALK_tSTIM_; Placebo + WALK_tSTIM_; and tAIH + WALK_tSHAM_. Participants receive daily tAIH (fifteen 90-sec episodes at 10% O_2_ with 60-sec intervals at 21% O_2_) or daily placebo (fifteen 90-sec episodes at 21% O_2_ with 60-sec intervals at 21% O_2_) before a 45-min session of WALK_tSTIM_ or WALK_tSHAM_. Our primary outcome measures assess walking speed (10-Meter Walk Test), endurance (6-Minute Walk Test), and balance (Timed Up and Go Test). For safety, we also measure pain levels, spasticity, sleep behavior, cognition, and rates of systemic hypertension and autonomic dysreflexia. Assessments occur before, during, and after sessions, as well as at 1, 4, and 8 weeks post-intervention. Results from this study extend our understanding of the functional benefits of tAIH priming by investigating its capacity to boost the neuromodulatory effects of transcutaneous spinal stimulation on restoring walking after SCI. Given that there is no known cure for SCI and no single treatment is sufficient to overcome walking deficits, there is a critical need for combinatorial treatments that accelerate and anchor walking gains in persons with lifelong SCI.

**Trial Registration:**

ClinicalTrials.gov, NCT05563103.

## Introduction

Walking deficits limit functional independence and increase comorbidities in nearly all persons with chronic (>1 year) spinal cord injury (SCI). Restoring the capacity for overground walking (e.g., speed and endurance) lowers healthcare costs for patients, their families, and caregivers and improves independence and quality of life. These are compelling reasons why reducing deficits in walking remains a high priority for many with SCI.^1^ Even minor improvements in walking that enable a person to walk independently within their community safely and efficiently may translate into significant health advantages. Given that there is no known cure for SCI and no single treatment is sufficient to overcome walking deficits, there is a tremendous need for targeted therapies that boost walking recovery for persons living with chronic SCI.

Combining treatments that harness endogenous mechanisms of plasticity shows promise in improving functional recovery after SCI. In particular, repetitive exposures to modest bouts of low oxygen (therapeutic acute intermittent hypoxia; tAIH) elicit remarkable neural plasticity through *de novo* synthesis of brain-derived neurotrophic factor (BDNF)-dependent signaling cascades^2^ that strengthen synaptic pathways to respiratory motor neurons in rodent SCI models.^3,4^ Lovett-Barr and colleagues showed that daily (7 consecutive days) tAIH before ladder walking practice improved forelimb stepping and breathing capacity in rodents with chronic, incomplete SCI.^5^ Since then, several randomized controlled trials showed that repetitive (up to 14 sessions) tAIH elicited similar training-related improvements in walking in persons with chronic (>6 months), incomplete SCI.^6–8^ Hayes and colleagues found that 5 consecutive days of tAIH + WALK improved overground walking endurance, but tAIH alone did not.^6^

These studies provide a conceptual framework to support the possibility that tAIH primes residual spinal circuitry after SCI and “boost” the functional benefits of task-specific training. Using tAIH as a primer for other promising treatments that target similar mechanisms of spinal plasticity may help overcome constraints on the rate and duration of motor recovery after chronic SCI.

tAIH may enhance the neuromodulatory effects of transcutaneous spinal stimulation (tSTIM) on restoring walking after SCI. tSTIM may engage spared afferent pathways and descending supraspinal signaling to excite spinal interneurons and motor neurons closer to their firing threshold.^9^ Clinical case reports involving persons with severe paralysis show that epidural electrical stimulation can elicit voluntary muscle activity and bipedal stepping.^10–12^ Concurrent application of repetitive (2 weeks) tSTIM combined with skill-based walking practice (WALK_tSTIM_) enhanced training-related gains.^13–15^ However, these studies showed that many of the reported improvements in overground walking required months of training to achieve functionally meaningful gains. One possible explanation is that plasticity wanes with time, such that those with chronic SCI experience limited training-related improvements.^16^

We speculate that repetitive tAIH and WALK_tSTIM_ act synergistically, so the combination of these modalities may further enhance training-related gains in walking function. Although the exact neural mechanisms and dosing for tAIH- and tSTIM-induced facilitation in walking performance are not fully understood in humans, there is ample pre-clinical evidence that repetitive exposures to these therapies trigger rapid changes in functional connectivity within and between the brain and spinal cord structures.^4,17,18^ Thus, this clinical trial protocol aims to explore the safety and efficacy of tAIH pre-treatment to WALK_tSTIM_ in restoring functional walking for persons with chronic SCI.

### Hypothesis and aims

**FIG. 1.**
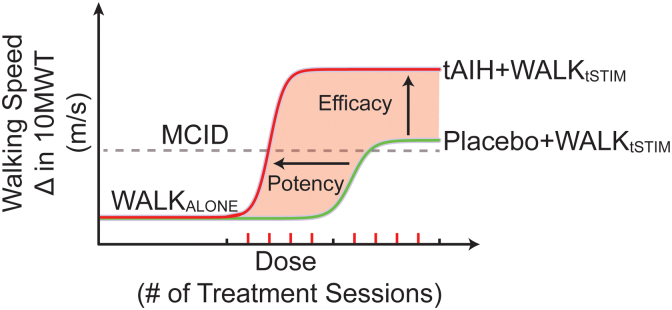
Illustration of study hypotheses. We hypothesize that the combination of tAIH + WALK_tSTIM_ will result in larger, more persistent improvements (increase in efficacy) in walking speed than tAIH + WALK_tSHAM_ or Placebo + WALK_tSTIM_. Additionally, we hypothesize that tAIH + WALK_tSTIM_ will result in fewer training sessions to achieve an MCID in walking speed improvements (greater potency) compared to Placebo + WALK_tSTIM_ or tAIH + WALK_tSHAM_. MCID, minimal clinically important difference; tAIH, therapeutic intermittent hypoxia; WALK_tSTIM_, transcutaneous spinal stimulation-enhanced walking therapy.

This study aims to expand tAIH's therapeutic “reach” as a pre-treatment for restoring function in persons with chronic SCI. Specifically, we propose to combine tAIH, transcutaneous spinal stimulation, and task-specific training to maximize function in spared neural pathways. Using an established tAIH protocol known to elicit improvement of walking ability in persons with chronic spinal injuries,^6^ we first propose to investigate the potential of tAIH (2 weeks) as a pre-treatment to WALK_tSTIM_ to elicit faster (potency) and persistent gains (efficacy) in walking for persons with chronic SCI (Fig. 1). We hypothesize that tAIH + WALK_tSTIM_ will boost the enduring effects of training-related walking improvements in persons with chronic SCI (Aim 1, efficacy). We also aim to determine the minimal number of combinatorial sessions needed to achieve a minimal clinically important difference (MCID) in walking improvements (Aim 2, potency) without eliciting maladaptive changes (Aim 3, safety).

These aims will be the first to investigate the safety and efficacy of tAIH priming with WALK_tSTIM_ to augment walking recovery in persons with chronic SCI. Determining how to harness these potentially synergistic treatments may lead to meaningful recovery that profoundly impacts the lives of persons with SCI while establishing more potent therapeutic strategies as a part of “best practices in SCI care.”

## Methods

### Study design

In this double-blinded, placebo-controlled, randomized controlled trial (ClinicalTrials.gov identifier: NCT05563103), we are examining the efficacy, potency, and safety of repetitive tAIH before WALK_tSTIM_ in a cohort of adults with chronic SCI in two tertiary hospitals: Spaulding Rehabilitation Hospital (Cambridge, MA) and Shirley Ryan AbilityLab (Chicago, IL). The Mass General Brigham (MGB) Institutional Review Board (IRB) approved the study protocol following the Declaration of Helsinki. Recruitment and enrollment of the study commenced in January 2023.

### Sample size

**FIG. 2.**
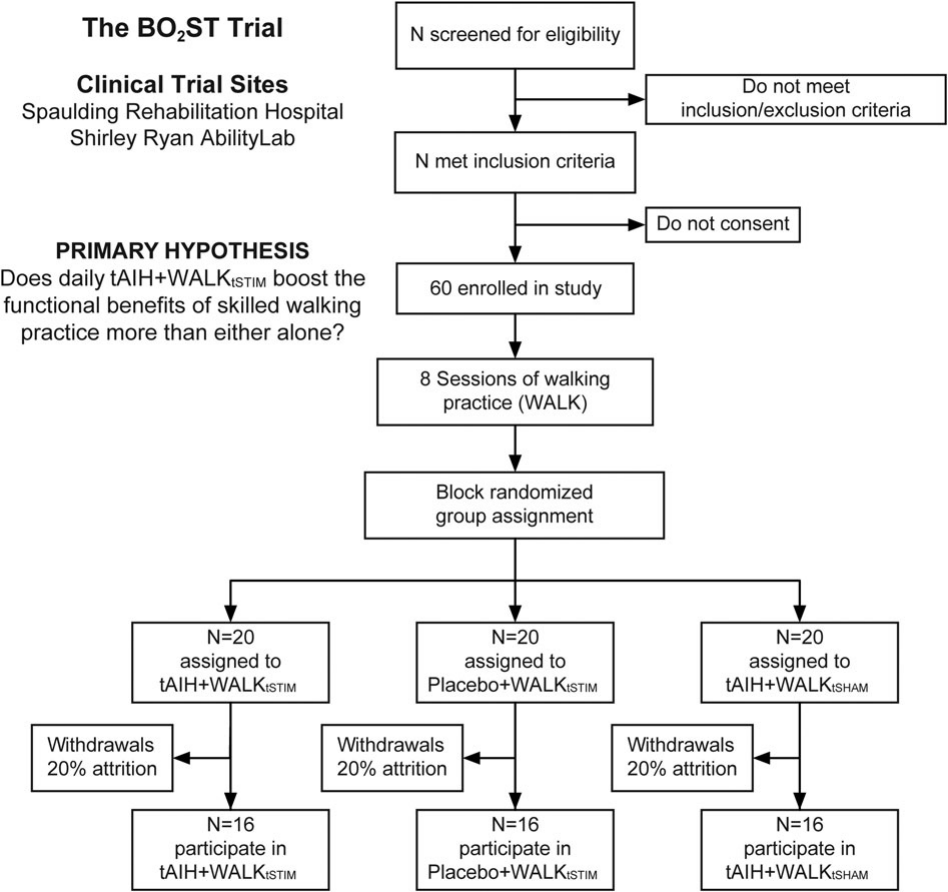
Flow diagram of anticipated subject recruitment, enrollment, intervention allocation, and disposition status. We anticipate *N* = 48 people with SCI to complete the study. SCI, spinal cord injury; tAIH, therapeutic intermittent hypoxia; WALK_tSTIM_, transcutaneous spinal stimulation-enhanced walking therapy.

We plan to enroll *N* = 60 persons with chronic, incomplete SCI in this clinical trial. We created a Consolidated Standard of Reporting Trials (CONSORT) flow diagram (Fig. 2) to summarize our trial enrollment, intervention, allocation, follow-up, and analyses.^19^ The initial sample size is an estimate from conservative power calculations on preliminary data that support our hypotheses testing in Aims 1 and 2.

For Aim 1, our sample-size estimate focused on results from an earlier placebo-controlled clinical trial that reported that persons with incomplete SCI completed the 10-Meter Walk Test (10MWT) faster after tAIH + WALK (−4.3 ± 1.0 sec, faster relative to baseline) as compared to Placebo + WALK (0.9 ± 0.4 sec, slower relative to baseline).^6^ From these results, we conservatively estimated the standard deviation of the 10MWT time relative to baseline as 2.0 sec in Placebo + WALK_tSTIM_ and tAIH + WALK_tSHAM_ and 3.0 sec in tAIH + WALK_tSTIM_. The estimated difference between the change in tAIH + WALK_tSTIM_ and the change in Placebo + WALK_tSTIM_ was −5.2 sec, and that difference between tAIH + WALK_tSTIM_ versus tAIH + WALK_tSHAM_ was estimated as −3.14 sec. Assuming a 20% dropout rate with 16 completers per group, three outcome measures (10MWT, Timed Up and Go Test [TUG], and the 6-Minute Walk Test [6MWT]), and a significance level of 0.0167 adjusted for the three repeated outcomes,^20^ this study will have >99% statistical power in comparing tAIH + WALK_tSTIM_ versus Placebo + WALK_tSTIM_ and >81% statistical power in comparing tAIH + WALK_tSTIM_ versus tAIH + WALK_tSHAM_.

For Aim 2, our sample-size estimate focused on results from a preliminary crossover study (unpublished) that showed that *N* = 3 people with incomplete SCI improved their walking speed after 5 consecutive days of tAIH + WALK_tSTIM_. Two participants achieved an MCID on their 10MWT at post-treatment day 5. However, no participants achieved an MCID after 5 consecutive days of Placebo + WALK_tSTIM,_ where the maximum increase in speed corresponded to 0.03 m/s. We predict that participants will require at least seven sessions of Placebo + WALK_tSTIM_ to achieve an MCID in walking speed. Using bootstrap sampling with replacement, we estimated the standard deviation for the number of sessions to achieve an MCID from our pilot study to be 1.5 days. Assuming that seven Placebo + WALK_tSTIM_ sessions are needed in order to achieve MCID as compared to five tAIH + WALK_tSTIM_ sessions estimated from our pilot data, the statistical power is >95% to detect the group difference to achieve an MCID on the 10MWT, with the sample sizes of 16 per group after 20% dropout.

### Randomization

Before enrolling the first study participant, a research statistician generated the balanced randomization scheme for treatment allocation at both clinical sites using the R Statistical Package.^21^ This method randomly assigns participants to one of three intervention groups (*N* = 20 per group): 1) tAIH + WALK_tSTIM_, 2) tAIH + WALK_tSHAM_, and 3) Placebo + WALK_tSTIM_ in blocks of 6 participants. Using this scheme, the site coordinator will assign each participant to their respective intervention group in the order in which they complete the walking practice sessions. Although the coordinator informs participants about their possible group assignment, they will be blinded to the treatment they receive (i.e., tAIH or Placebo, WALK_tSTIM_, or WALK_tSHAM_). The clinical raters and trainers will also be unaware of each participant's treatment assignment. Based upon reports from participants blinded during our previous tAIH study,^6^ >75% guessed incorrectly or were uncertain of the breathing intervention received, suggesting that our previous blinding and air delivery methods were effective.

We plan a similar integrity analysis for the stimulation sham protocols (no stimulation). Participants guess the interventions received at the end of each treatment session and indicate their guess confidence using a Likert scale.^22,23^ Using a contingency table and Fisher's exact test, we will determine whether the probability of correct guessing differs from chance.

### Study recruitment and enrollment

We established an interprofessional recruitment team at Spaulding Rehabilitation Hospital and Shirley Ryan AbilityLab. The recruitment team comprises the site principal investigators, clinical trial coordinators, research assistants, post-doctoral research fellows, onsite physicians, and research physical therapists. Our study recruitment process includes reviews of site-specific hospital patient registries, announcements through clinical research networks (e.g., Spinal Cord Injury Model Systems), clinicaltrials.gov, outreach to local outpatient rehabilitation centers, and media postings on relevant websites. Our team clinicians will also contact patients with SCI diagnoses admitted to our clinical sites that will likely meet the study criteria once they reach the chronic stage of injury. The study personnel contact potential participants using IRB-approved scripts to avoid bias or coercion during recruitment. Study participants must meet the study inclusion and exclusion criteria detailed in Table 1. These criteria align with the International Campaign for Cures of Spinal Cord Injury Paralysis recommendations^24^ and account for ethical considerations, safety, and potential confounds during participant recruitment.

**Table 1.** Study Eligibility Criteria

Inclusion criteria
• 18–70 years of age
• Medically stable with medical clearance from study physician to participate
• Incomplete SCI at and between levels C_2_ and L_2_ with at least some sensory or motor function preserved below the neurological level to ensure phrenic and cauda equina sparing
• Non-progressive etiology of SCI
• ISNCSCI Impairment Scale,^69^ Grade C–D
• Ambulatory, able to walk 10 meters without support from another person
• Chronic injury (≥12 months post-injury)^a^
Exclusion criteria
• Severe concurrent illness or pain^b^
• Less than a score of 24 on the MMSE^c^
• Severe recurrent autonomic dysreflexia per assessment by the study physicians
• History of severe cardiovascular/pulmonary complications, including hypertension (systolic blood pressure >150 mm Hg)
• Pregnancy^d^
• Botulinum toxin injections in lower extremity muscles within the last 6 months
• History of tendon or nerve transfer surgery in the lower extremity
• Untreated severe sleep-disordered breathing^e^
• Active implanted devices (e.g., intrathecal baclofen pump)
• Receiving electrical stimulation therapy
• tSTIM-induced involuntary motor threshold is >200 mA (exceeding SCONE's current limit).

^a^ Chronic SCI to avoid the potential for spontaneous neurological plasticity and recovery.^16,70^

^b^ Illness including unhealed decubiti, severe neuropathic or chronic pain syndrome, severe infection (e.g., urinary tract), hypertension, cardiovascular disease, pulmonary disease, severe osteoporosis, active heterotopic ossification in the lower extremities, and severe systemic inflammation.

^c^ MMSE^25^ cutoff to avoid participants with cognitive impairment.

^d^ Pregnancy is an exclusion criterion because of unknown effects of tAIH or tSTIM on a fetus; persons of childbearing potential will not otherwise be excluded.

^e^ Severe sleep-disordered breathing (>30 apneas or hypopneas per hour).^26^

ISNCSCI, International Standards for Neurological Classification of Spinal Cord Injury; MMSE, Mini-Mental State Examination; SCI, spinal cord injury; tAIH, therapeutic intermittent hypoxia; tSTIM, transcutaneous spinal stimulation;

Potential participants undergo a medical screen by the study physicians and a physical/mental evaluation by the clinical research team. Eligible persons are informed of the study procedures and potential risks and sign the IRB-approved informed consent form before participation. The screening consists of a medical record review, assessment of the participant's ability to walk 10 m without human assistance, confirmation that their lower extremity involuntary motor threshold is <200 mA, and Mini-Mental State Examination (MMSE) score ≥24.^25^ We also screen for sleep-disordered breathing using a portable sleep monitor (Nox A1s PSG System; Nox Medical, Suwanee, GA or ApneaLink; ResMed Inc., San Diego, CA) at home for 1 night.^26^ After the screening, we deidentify study participants with a unique alpha-numeric identifier and associate this code with study-related documentation. This study received approval from the MGB IRB (#2022P002036) and the Department of Defense Office of Human Research Oversight in January 2023, and the first study participant enrolled in March 2023.

### Experiment protocol

**FIG. 3.**
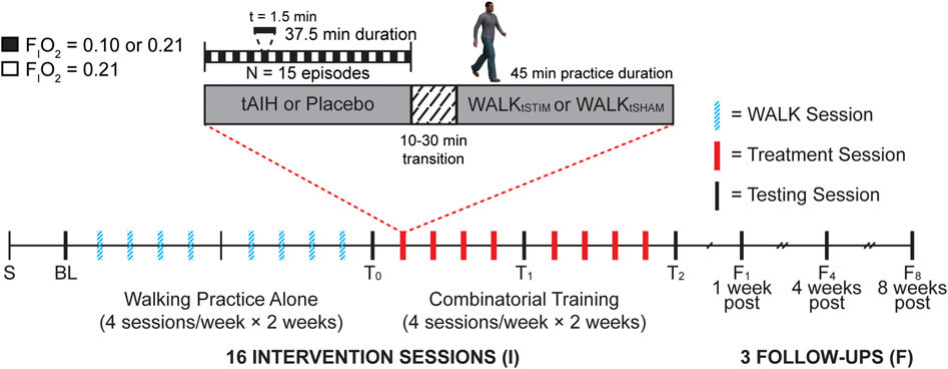
Timeline of interventions and assessments. After the initial screening visit (S), each intervention consists of eight sessions of walking training (WALK) alone, followed by eight additional sessions of tAIH or Placebo before WALK_tSTIM_ or WALK_tSHAM_. Assessments occur at baseline (BL), at the end of intervention weeks 2 (T_0_), 3 (T_1_), and 4 (T_2_), and, again, after 1 (F_1_), 4 (F_4_), and 8 weeks (F_8_) after the last intervention session. tAIH, therapeutic intermittent hypoxia; WALK_tSTIM_, transcutaneous spinal stimulation-enhanced walking therapy.

We developed a study protocol to measure the extent to which tAIH priming boosts the efficacy and potency of training-related functional gains in persons with chronic SCI (Fig. 1). Study participants enroll in 23 intervention and assessment sessions at one of the two clinical trial sites (Fig. 3). Physical therapists serve as trainers and raters; they complete competency and protocol training before their study involvement to ensure rater and training reliability within and between the clinical sites. The interventions consist of eight sessions of walking practice followed by eight combinatorial treatment sessions (four sessions per week for 4 consecutive weeks).

We anticipate that tAIH before WALK_tSTIM_ will exceed the benefits of walking practice (WALK), tAIH + WALK_tSHAM_, or Placebo + WALK_tSTIM_ on enhancing the performance of the trained task of overground walking. Justification for the treatment sequence of tAIH followed by WALK_tSTIM_ is based on the estimated time (minutes) required for tAIH to facilitate expression of BDNF.^27^ Applying tAIH ∼1 h before WALK_tSTIM_ may promote temporal alignment of mechanisms of WALK_tSTIM_ neuromodulation with complementary mechanisms of tAIH-induced plasticity that involve BDNF. Details on these interventions are below.

#### Walking practice

Participants receive eight 45-min WALK sessions over 2 consecutive weeks. The WALK sessions consist of intensive walking-related functional tasks using a skill-based training approach.^6^ The training includes activities related to walking speed (10MWT), initiation and balance (TUG), and walking endurance (6MWT). Recent studies found that skill-based walking practice is more consistent with community walking, providing meaningful gains in walking function after neurological injury.^28–31^ Walking practice emphasizes learning sensorimotor skills for community walking, including walking speed, endurance, stepping balance, and initiation.^30^ Participants may rest up to 15 min during the practice sessions. During the WALK sessions, the physical therapist trainer will provide physical assistance as needed for safety. Step count, peripheral blood oxygen saturation (SpO_2_), heart rate (HR), and blood pressure (BP) are measured at each session.

#### Therapeutic acute intermittent hypoxia (tAIH/Placebo)

**FIG. 4.**
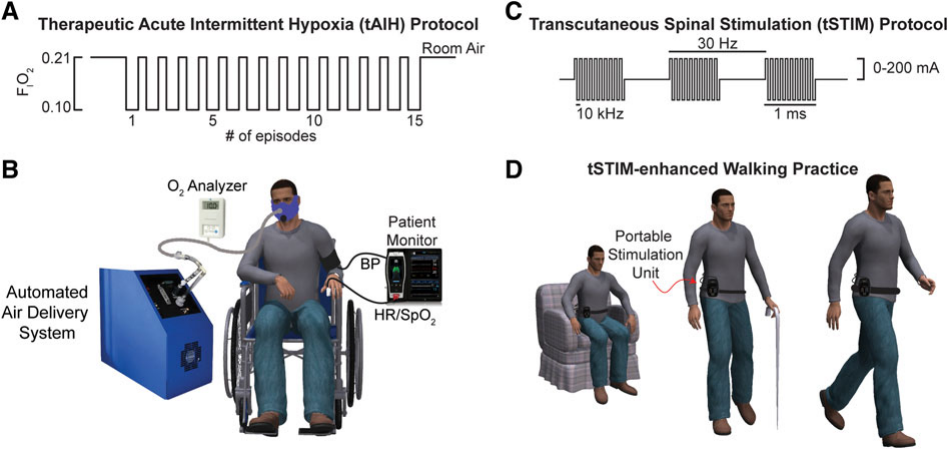
Illustration of intervention protocols (**A**) A participant receives a tAIH sequence consisting of fifteen 1.5-min episodes of breathing low oxygen (10% O_2_) with 1-min intervals of breathing room air (21% O_2_). (**B**) We monitor the participant's heart rate (HR), blood oxygen saturation (SpO_2_), and blood pressure (BP) during tAIH intervention. (**C**) During the tSTIM protocol, participants receive a sequence of 1-ms bursts of electrical stimulation at 30 Hz with a 10-kHz carrier frequency that targets the thoracolumbar region (T_11_–L_2_). We set the stimulation amplitude to a subthreshold intensity between 0 and 200 mA before the intervention session (**D**). The subthreshold stimulation is active during 45 min of skill-based walking practice (WALK_tSTIM_). tAIH, therapeutic intermittent hypoxia; tSTIM, transcutaneous spinal stimulation; WALK_tSTIM_, transcutaneous spinal stimulation-enhanced walking therapy.

Each participant receives eight ∼40-min tAIH sessions (2 weeks, four sessions per week) of tAIH or sham. A tAIH session consists of 15 episodes of 90-sec breathing low oxygen (F_I_O_2_ = 0.10 ± 0.02, i.e., 10% O_2_) or breathing room air (F_I_O_2_ = 0.21 ± 0.02, i.e., 21% O_2_) for sham with 60-sec intervals of room air (Fig. 4). We provide the treatments by a custom automated air delivery system as previously described.^32^ In brief, the delivery system alternately directs an air from a pressure-swing adsorption low-oxygen generator (HYP-123; Hypoxico Inc., New York, NY) and a flow-matched blower source to the non-rebreather face mask. We monitor and record SpO_2_ and HR at 1-sec intervals and BP every fifth breathing interval using a vital monitor (Masimo Root, Radical-7; Masimo Corp., Irvine, CA).

#### Spinal stimulation during walking practice (WALK_tSTIM_/WALK_tSHAM_)

Participants receive eight 45-min sessions (2 weeks, four sessions per week) of either the combination of WALK and transcutaneous electrical stimulation (WALK_tSTIM_) or WALK and transcutaneous sham stimulation (WALK_tSHAM_). A research team member, not blinded to the intervention, configures the portable stimulation system (SCONE; SpineX Inc; Los Angeles, CA; Fig. 4C,D) and places the device superficial to the thoracolumbar region of the spinal cord. The stimulating electrodes are placed at the midline on the trunk superficial to the spinous processes of the T_11_–T_12,_ and L_1_–L_2_ vertebrae.^33,34^ The grounding electrodes are placed over the left and right iliac crests. The device can deliver mono- and biphasic current with a 1-ms pulse width at a 30-Hz base frequency and a 10-kHz carrier frequency. However, a biphasic waveform is preferred for delivering high-amplitude stimulation with minimal discomfort.^17,35^ This is likely attributable, in part, to the biphasic waveform's capacity to attenuate unmyelinated c-fibers activity and to penetrate deeper because of lower tissue impedance.^36^ During the WALK_tSTIM_ conditions, the current is set to 80% of the motor threshold, as detailed previously,^33^ and 0 mA during the WALK_tSHAM_ condition. The motor threshold corresponds to the minimum stimulation intensity to achieve an involuntary motor activity in the lower extremities.

#### Concomitant medication

Medication use (e.g., bowel/bladder dysfunction, systemic hypertension, pain, spasticity, and antidepressant medications) is common after SCI and may confound the effects of our interventions. Because of participant safety and comfort, we will not limit prescribed medication use. We will require those receiving medication to maintain the dose 1 week before and during the intervention sessions, as we did in our previous study.^6,8^ The team physicians work with the study participant to ensure that medication dosing is stable before enrollment and during interventions. We log any change in medication for each participant throughout the study and assess for possible drug-tAIH interactions and changes in prescribed drug dosages at follow-ups. If participants must change their medications for safety, they can readily withdraw from the study at any time.

### Safety precautions

A designated research monitor (medical doctor) will oversee the safety of the research and report observations and findings to the IRB. This includes a review of unanticipated problems involving risks to participants or others and an independent report. To ensure participant safety, we implement the following precautions.

#### Protection against fall risk

Participants complete the walking training and assessments with a physical therapist to minimize fall risk. Participants also may use their least restrictive powerless assistive device. Participants will be asked about their fall history during the screening process, and any adverse events (falls) will be documented during the intervention.

#### Protection against fatigue risk

Assessment sessions may last up to 3 h. Only 60–90 min are dedicated to physical assessments, but this duration may be fatiguing to some. Most of the allotted time is devoted to subject preparation and instrumentation, done while the subject is sitting or lying down. During assessments, the evaluator and trained assistants monitor the participant for signs of fatigue and instability.

#### Protection against cardiopulmonary risk

During interventions, a team member monitors cardiopulmonary function to ensure safety and comfort. If excessive discomfort or pain is reported, the intervention ends. In case of medical issues that may arise during our interventions, such as symptoms of autonomic dysreflexia (AD), rapid change in HR or systolic BP (SBP), diaphoresis, severe headache, or dizziness, participants will be withdrawn from the experiment and provided skilled care from the research clinicians. The emergency medical team at the clinical sites are notified promptly. Return to the study requires medical clearance.

#### Protection against low oxygen risks

Moderate reduction in inspired oxygen can cause a lightheaded sensation, dizziness, reduced vision, and euphoria. The research team monitors SpO_2_, HR, and BP before, during, and after the breathing intervention to ensure that administered oxygen levels do not induce cardiopulmonary stress on participants. The breathing system automatically delivers normal room air to participants if their SpO_2_ level falls below 75%. Supplemental oxygen will be available for participants if needed. If participants report signs of a lightheaded sensation, dizziness, reduced vision, and euphoria, the experiment will be stopped, administration of room air or supplemental oxygen will be provided, and a physical therapist and/or physician will reassess the participant.

#### Protection against electrical shock

Risk of electrical shock is extremely low, and the SCONE device is limited to non-injurious current and voltage levels. The electrical stimulation device is routinely checked and constantly monitored for defects. Electrodes are checked before each session to maintain quality assurance. The emergency medical teams at the trial sites will be notified promptly if any electrical shocks occur.

#### Protection against skin irritation

Some subjects are sensitive to the hydrogel electrodes or adhesive used in medical-grade tape. This can result in temporary redness or itchiness in areas where tape has contacted the skin. We will ask subjects if they have known tape or adhesive sensitivity before the experiment begins. We use hypoallergenic medical-grade tapes and adhesives commonly used in hospitals.

#### Protection of confidential information

For participant confidentiality, all data will be coded and unrelated to identifiable information. Participants' names will only appear on the research consent form and not on any data sheets or files kept in the laboratory. Names or other information that could be used to identify persons will not appear when the study results are presented or published. However, video files will be created to serve as a visual record of the experiment, and they cannot be changed to disguise the participant's appearance. All research records, including video records, will be kept on computers accessible only to study staff or in locked archives.

Only study investigators, the investigator's staff, site IRBs, and the sponsor's Office of Human Research Oversight will have the authority to review the study records; they must maintain the confidentiality of each participant's identity. Records of ongoing participation in this study remain confidential and on Health Insurance Portability and Accountability Act–compliant computer systems or in a secure file cabinet in the site research offices. All research materials remain confidential until the study is completed, at which point all subject-sensitive research material will be destroyed. Results of this study may be used for publications or presentations at scientific meetings; if individual results are discussed, the participant's identity will be protected using a study identification number rather than identifying information.

### Outcome measures

We provide a summary of primary and secondary outcome measures in Table 2.

**Table 2.** Frequency of Clinical Assessments and Outcome Measurements

Measurement	Data collected	Time collected
Functional assessments		
Demographics	Age, sex, ethnicity, race, education	S
Medical history	SCI characteristics, medical records	S
Sleep quality^26^	Apnea-hypopnea rate	S
Medications	Name, dose, duration, and purpose	S, B, T, F
10-Meter Walk Test^71^	Time (s)	B, I, T, F
6-Minute Walk Test^72^	Distance (m)	B, T, F
Timed Up and Go Test^73^	Time (s)	B, T, F
WISCI II^74^	Scalar score (0–20)	B, T, F
SCI-FAI^41^	Scalar score (0–39)	B, T, F
Physiological assessments		
ISNCSCI exam^69,a^	SCI classification grade (A–E)	S
LEMS^42^	Scalar score (0–50)	B, T, F
SCATS^75^	Scalar score (0–18)	B, T, F
NPRS^43,44^	Scalar score (0–10)	B, T, F
NBDS^54^	Scalar score (0–47)	B, T, F
NBSS^55^	Scalar score (0–78)	B, T, F
Cardiovascular measures	Blood pressure, heart rate, SpO_2_, respiratory rate	B, I, T, F
Neurocognitive assessments		
MMSE^25^	Memory score (0–30)	S
CVLT3^49^	Total recall index score (40–160)	T
O-TMT^50^	Duration (s)	T

S = screening day; B = baseline; T = testing session; F = follow-up (1, 4, and 8 weeks); I = intervention day.

^a^ If no current/recent record of ISNCSCI exam is available.

CVLT3, California Verbal Learning Test, Third Edition; ISNCSCI, International Standards for Neurological Classification of Spinal Cord Injury; LEMS, lower extremity motor score; MMSE, Mini-Mental State Examination; NBDS, Neurogenic Bowel Dysfunction Score; NBSS, Neurogenic Bladder Symptom Score; NPRS, Numeric Pain Rating Scale; O-TMT, Oral Trail Making Test; SCATS, SCI Assessment Tool for Spastic Reflexes; SCI, spinal cord injury; SCI-FAI, SCI Functional Ambulation Inventory; WISCI, Walking Index for Spinal Cord Injury.

#### Gait

Overground walking ability is quantified by three primary measures: 10MWT (walking speed); TUG test (walking initiation and balance); and a 6MWT (walking endurance). These tests capture functional ambulation, exhibit high reliability and construct validity, and have precedence for quantifying and distinguishing degrees of functional ambulatory recovery post-SCI.^37–39^ Our previous study in persons with chronic SCI showed these tests to be more sensitive to changes in walking function and functional ambulation compared to American Spinal Injury Association Impairment Scale (AIS) grade or categorical ambulation metrics.^6,8^ During all walking assessments, we allow participants to use their least restrictive hand-held assistive device of choice.

Participants carry out two trials each for the 10MWT and TUG (at their fastest but comfortable and safe speed); the average speed will be used. A minimum of a 1-min rest between tests will be provided. Participants will perform the 6MWT only once at a walking speed sustainable for 6 min. The participant's walking distance at the 2- and 6-min mark will be recorded. We also record secondary outcome measures of walking function that include the Walking Index for Spinal Cord Injury (WISCI) II^40^ and SCI Functional Ambulation Index (SCI-FAI).^41^ To quantify strength, the AIS lower extremity motor scores (LEMSs) will be used.^42^

To quantify maladaptive changes that may occur subsequent to the combinatorial interventions, we measure pain, spasticity, systemic hypertension, AD, and cognitive ability during our assessments.

#### Pain severity

Pain severity is assessed using the 11-point Numeric Pain Rating Scale (NPRS) of 0 (no pain) to 10 (extreme pain). The NPRS has shown high test-retest reliability and interpretability as well as good content and construct validity for persons with SCI.^43,44^

#### Spasticity

Spasticity is assessed using rhe Spinal Cord Assessment Tool for Spastic Reflexes (SCATS)^45^; three subscales will be used to quantify spasticity in lower extremity clonus, flexor, and extensor spasticity.

#### Blood pressure

We will define a systemic hypertensive event as a systolic pressure exceeding 140 mm Hg and/or diastolic pressure exceeding 90 mm Hg.^46^ For each participant, we specifically record the hypertension incident rate, that is, the number of hypertensive events divided by units of person-measures (the total number of BP measurements), which accounts for the total number of chances for detecting a hypertensive event and for measurements not made because of dropout or a disqualifying adverse event.^47^

#### Autonomic dysreflexia incidence rate

AD incidence rate is the number of AD events divided by the total person-time (number of study days completed by each participant) to account for the total number of chances for detecting AD for days not measured because of dropout or a disqualifying adverse event.^47^ An AD event will constitute a participant having a SBP increase from baseline of 20 mm Hg not associated with exercise or SBP >150 mm Hg with complaints of headache, diaphoresis, and/or blurred vision and will be diagnosed by our study team clinicians (physicians and physical therapists).

#### Cognitive ability

We will assess cognitive function after the last walking practice session and after the last intervention session (T_0_, T_2_; Fig. 3).^48^ The California Verbal Learning Test, Third Edition (CVLT3) brief form will quantify verbal learning and memory.^49^ We also assess executive function with the Oral Trail Making Test (O-TMT).^50^ These tests examine the areas of cognitive ability most likely to be affected by an excessive dose of low oxygen, such as during severe sleep apnea.^51^

#### Bowel and bladder function

We qualitatively assess bowel and bladder function using standardized self-reported questionnaires: the Neurogenic Bowel Dysfunction Score (NBDS) version 2.1 and the Neurogenic Bladder Symptom Score (NBSS).^52–55^ These questionnaires show high test-retest reliability and interpretability.

### Statistical analysis

We plan to test three subhypotheses using parametric and non-parametric statistical inference methods. Statistical significance corresponds to a *p* value <0.05.

#### Hypothesis 1

Daily tAIH + WALK_tSTIM_ enhances overground walking ability in persons with chronic SCI compared to tAIH + WALK_tSHAM_ or Placebo + WALK_tSTIM_.

We predict a decrease in 10MWT and TUG time and an increase in 6MWT distance relative to baseline after tAIH + WALK_tSTIM_ (vs. Placebo + WALK_tSTIM_, tAIH + WALK_tSHAM_). We quantify walking ability using three outcome measures: change in TUG, 10MWT, and 6MWT relative to baseline assessments. Change in performance on these measures will occur after the last intervention session and again at 1-, 4-, and 12-week post-intervention follow-ups (Fig. 3). We will test three related subhypotheses using a linear mixed model with intervention (tAIH + WALK_tSTIM_, tAIH + WALK_tSHAM_, and Placebo + WALK_tSTIM_) and time (visit number) as the fixed main effects, with subject as random effect, whereas scores for TUG (time), 10MWT (time), and 6MWT (distance) will be considered as repeated measures.^56^

Differences from assessment after completion of the walking practice sessions will be compared between and within interventions at tests (T_1_, T_2_) and follow-ups (1, 4, and 8 weeks post-intervention). If analyses of variance (ANOVAs) reveal significant differences, the Tukey-Kramer *post hoc* test will identify pair-wise differences. If walking performance measures at the end of the walking practice sessions are significantly different between intervention groups, we will use an analysis of covariance. We also plan to fit a linear mixed model to test the magnitude and duration of tAIH priming effects based on linear contrasts after controlling for the study period, baseline primary outcomes, and demographics data.

#### Hypothesis 2

Daily tAIH priming enhances the potency of WALK_tSTIM_ on improving walking speed in people with chronic SCI. We quantify potency as the number of tAIH + WALK_tSTIM_ sessions (eight total) needed to achieve the MCID in walking speed.^57^ We assess walking speed with the 10MWT at the end of each training session and predict that those who receive tAIH + WALK_tSTIM_ will achieve an MCID with fewer training sessions than either treatment alone (Placebo + WALK_tSTIM_ or tAIH + WALK_tSHAM_). To test this hypothesis, we will use linear mixed models with number of sessions to achieve the MCID as the outcome with treatment group and 10MWT speed at baseline as fixed effects and participant as the random effect. We anticipate that improvement in walking recovery also will correlate with improved 6MWT, TUG, SCI-FAI, WISCI II, and LEMS scores, suggesting the clinical relevance of this intervention across impairment levels and walking deficits.

#### Hypothesis 3

Daily tAIH+WALK_tSTIM_ does not induce maladaptive changes (spasticity, pain, systemic hypertension, and AD) in persons with chronic SCI. We predict no difference in SCATS, NPRS, CVLT, O-TMT, NBDS, and NBSS scores after daily tAIH + WALK_tSTIM_ compared to baseline. We will use a Friedman two-way repeated-measures ANOVA by ranks to compare these relative scores between and within groups. Changes in these scores relative to baseline are the repeated measures. Intervention (tAIH + WALK_tSTIM_, Placebo + WALK_tSTIM_, and tAIH + WALK_tSHAM_) and time are the fixed main effects, with subject as the random effect. We also predict that tAIH + WALK_tSTIM_ will not elicit a greater incidence of systemic hypertension or AD in persons with chronic SCI.

To test these subhypotheses, we will compare the incidence rates of these maladaptive changes between interventions using the relative risk.^58,59^ A relative risk of 1 will indicate no association between the maladaptive measures and interventions. Using the 95% confidence interval of the relative risk,^59^ we will determine whether there is a statistically significant association between interventions. We predict that the relative risk of hypertension and AD are not different between tAIH versus placebo interventions and WALK_tSTIM_ versus WALK_tSHAM_ interventions.

To minimize biases and errors in data collection, a designated lead physical therapist, blinded to study interventions, ensures participant adherence to the protocol and consistent scoring among therapists by regularly checking all assessment logs for adherence to standard clinical procedures. Non-blinded research staff members (including post-doctoral fellows, lab engineers, and research assistants) assist clinicians with training setup and clinical data collection. The study principal investigator oversees all study procedures and ensures the correct collection and documentation of data. If participants miss more than two walking practice sessions or intervention sessions, we will not conduct further assessments and exclude their data from analyses. However, if participants miss an assessment session, we record their data as “missing” at that time point.

### Adverse event reporting

The research team will record adverse events related to each participant from the time of enrollment to the last follow-up assessment visit, which include: 1) unintentional loss of balance (i.e., fall to the ground); 2) change in systolic pressure to >140 mm Hg and/or diastolic pressure exceeding >90 mm Hg^46,60^; 3) AD with SBP >150 or >20 mm Hg from baseline with complaints of headache, diaphoresis, and/or blurred vision; 4) musculoskeletal injury during/after walking training (i.e., sprain, fracture, etc.); 5) symptoms such as pain, soreness, numbness, or signs of injury (inflammation, blisters, etc.) during or immediately after training or on returning home; 6) hospitalization for any cause; and 7) death attributable to any cause. Serious and/or unexpected adverse events will be immediately reported to the MGB IRB.

### Study compensation

Participants receive $40 per visit and up to $300 in travel reimbursement.

### Study limitations

Clinical trials are necessary for the study of combinatorial interventions to treat SCI. However, the experimental protocols for these trials are often beset with study design challenges attributable, in part, to confounding treatment interactions, small sample size, short study duration, and SCI heterogeneity. The study of multiple treatments and their interactions requires the appropriate choice of controls to discriminate between the clinical outcome of the treatments alone, combined, or neither. Identifying the study groups is pivotal to trial success, but requires larger sample sizes. Because of challenges from the ethical perspective in SCI research with humans, we cannot include a control (no treatment) group in the present trial. We anticipate that some participants will receive additional therapy services upon study enrollment.

We will quantify the interactions between frequency/duration of these services and our primary outcome measures. The biostatistician also will use multi-variate statistical models to assess these and other potential confounds to our outcome measures. Though most studies continue for longer periods (e.g., 12 weeks),^61^ up to eight sessions of tAIH or WALK_tSTIM_ are sufficient to elicit improvements in walking speed in persons with SCI.^6,8,62^ To consider the potential long-term effects of tAIH + WALK_tSTIM_ on walking ability, we will follow up at 1, 4, and 8 weeks post-treatment.

Small sample sizes can undermine the translation of results to the larger heterogeneous patient population. We also acknowledge that balancing treatment groups of a heterogeneous SCI cohort will be a challenge. To address this obstacle, we will consider age, sex, reliance on walking aids,^63^ and injury level. Second, we do not yet know the potency of WALK_tSTIM_ and eight sessions may be considered too few to achieve MCID in the treatment groups. We chose eight sessions to remain pragmatic in treatment duration while aligning our study design to a recent placebo-controlled trial that found WALK_tSTIM_ induced clinically significant gains in walking speed within six sessions.^62^ We acknowledge that achieving MCID in eight sessions may not occur during treatments without tAIH. If this happens, we will compare groups using survival-type analyses to include those with no participants who achieved MCID.^64^ Third, medication changes are frequent in persons with SCI, which may alter neural plasticity.

While our proposed study will allow for changes in pharmacological regimens only after (not during) our intervention sessions, we will monitor medication dosing throughout the study and account for them as covariate factors in our statistical models. The statistical power computed from our preliminary data involving a heterogeneous cohort of SCI persons offers confidence that our estimated sample size will detect differences between interventions if they exist.

Several concomitant factors can weaken the interpretation of the study results. Concomitant medication used (e.g., for bowel/bladder dysfunction, systemic hypertension, pain, spasticity, and depressive symptoms) may confound the effects of tAIH intervention. The stability of the medication regimen prior to enrollment may limit confounding effects. Future clinical integration of this combinatorial treatment is possible based on the simplicity, low-cost, and non-invasiveness of administering tAIH and tSTIM. We anticipate that results from this trial will provide new understandings of combinatorial treatments to augment traditional physical therapy.

## Discussion

This study aims to examine the efficacy, potency, and persistency of daily tAIH priming to boost the training-related effects of WALK_tSTIM_ on walking recovery in persons with chronic SCI. Prior studies show that daily tAIH elicits rapid improvements in overground walking ability when used alone or as a combinatorial approach, compared to training alone.^6–8,65^ Indeed, restoring community walking is a top priority for persons living with SCI, as improvements in walking function enable them to participate more independently in a broad range of daily living activities and combat the deleterious effects of secondary health conditions. Traditional gait training approaches offer only modest recovery of walking function in persons with SCI.^66^ Thus, treatments that may trigger mechanisms of neural plasticity within spared spinal pathways in a safe and efficacious manner, regardless of ambulatory status, is a top priority in SCI medicine.^67,68^ Follow-ups with participants months after the intervention ends will allow us to analyze the longevity of walking recovery. We anticipate that outcomes from this study will provide new insights into the potential clinical utility of tAIH-based translational approaches to boost training-related gains in functional mobility after SCI.

## Data Availability

Final trial data are accessible to the study investigators. However, individual data requests should be made to the principal investigator. The research team plans to disseminate results through published manuscripts and presentations. We also plan to make information available through the Spinal Cord Injury Common Data Elements (CDE) standards developed through the collaboration of the International Spinal Cord Society, the American Spinal Injury Association, and the National Institutes of Health National Institute of Neurological Disorders and Stroke. Per request of the funding agency's Program Office, we will submit data for USAMRMC archiving in accordance with privacy policies of the USAMRMC and institutional review boards at the collaborating sites: Spaulding Rehabilitation Hospital and Shirley Ryan AbilityLab.

## Abbreviations Used

6MWT: 6-Minute Walk Test
10MWT: 10-Meter Walk Test
AD: autonomic dysreflexia
AIS: American Spinal Injury Association Impairment Scale
ANOVA: analysis of variance
BDNF: brain-derived neurotrophic factor
BP: blood pressure
HR: heart rate
IRB: institutional review board
LEMS: lower extremity motor score
MCID: minimal clinically important difference
MGB: Mass General Brigham
MMSE: Mini-Mental State Examination
NBSS: Neurogenic Bladder Symptom Score
NBDS: Neurogenic Bowel Dysfunction Score
NPRS: Numeric Pain Rating Scale
O-TMT: Oral Trail Making Test
SBP: systolic BP
SCATS: SCI Assessment Tool for Spastic Reflexes
SCI: spinal cord injury
SCI-FAI: SCI Functional Ambulation Inventory
SpO_2_: peripheral blood oxygen saturation
tAIH: therapeutic acute intermittent hypoxia
tSTIM: transcutaneous spinal stimulation
TUG: Timed Up and Go Test
WALK: walking practice
WALK_tSHAM_: WALK and transcutaneous sham stimulation
WALK_tSTIM_: transcutaneous spinal stimulation-enhanced walking therapy
WISCI: Walking Index for Spinal Cord Injury
